# The Impacts of Domestication and Agricultural Practices on Legume Nutrient Acquisition Through Symbiosis With Rhizobia and Arbuscular Mycorrhizal Fungi

**DOI:** 10.3389/fgene.2020.583954

**Published:** 2020-09-30

**Authors:** Ailin Liu, Yee-Shan Ku, Carolina A. Contador, Hon-Ming Lam

**Affiliations:** Centre for Soybean Research of the State Key Laboratory of Agrobiotechnology and School of Life Sciences, The Chinese University of Hong Kong, Hong Kong, China

**Keywords:** legume-microbe interaction, arbuscular mycorrhizal fungi, symbiotic nitrogen fixation, rhizobia, domestication, metabolic modeling, metabolic profiling

## Abstract

Legumes are unique among plants as they can obtain nitrogen through symbiosis with nitrogen-fixing rhizobia that form root nodules in the host plants. Therefore they are valuable crops for sustainable agriculture. Increasing nitrogen fixation efficiency is not only important for achieving better plant growth and yield, but it is also crucial for reducing the use of nitrogen fertilizer. Arbuscular mycorrhizal fungi (AMF) are another group of important beneficial microorganisms that form symbiotic relationships with legumes. AMF can promote host plant growth by providing mineral nutrients and improving the soil ecosystem. The trilateral legume-rhizobia-AMF symbiotic relationships also enhance plant development and tolerance against biotic and abiotic stresses. It is known that domestication and agricultural activities have led to the reduced genetic diversity of cultivated germplasms and higher sensitivity to nutrient deficiencies in crop plants, but how domestication has impacted the capability of legumes to establish beneficial associations with rhizospheric microbes (including rhizobia and fungi) is not well-studied. In this review, we will discuss the impacts of domestication and agricultural practices on the interactions between legumes and soil microbes, focusing on the effects on AMF and rhizobial symbioses and hence nutrient acquisition by host legumes. In addition, we will summarize the genes involved in legume-microbe interactions and studies that have contributed to a better understanding of legume symbiotic associations using metabolic modeling.

## Introduction

Major macronutrients such as nitrogen (N), phosphorus (P), and potassium (K) play important roles in crop development. Lack of soil fertility is a vital constraint on crop production. To avoid intensifying the use of chemical fertilizers which may cause irreversible environmental damages including soil salinization and eutrophication of local lakes and rivers from fertilizer runoffs, plant growth-promoting rhizobacteria (PGPR) are regarded as efficient biofertilizers (Singh et al., 2016). The application of biofertilizers to the soil increases the biodiversity in the soil, and improves soil fertility through N_2_ fixation, as well as P and K mobilization in the form of organic acids (Egamberdiyeva and Höflich, 2004; Sulieman and Tran, 2014; Itelima et al., 2018).

Legumes are important crops in agriculture, not only because they are a protein-rich food source, but also because of their contribution to soil fertilization through symbiotic nitrogen fixation (SNF) as a result of their association with nitrogen-fixing PGPR, i.e., rhizobia (Zahran, 1999). Each year, legumes can provide more than 70 million tonnes of N to soil (Brockwell et al., 1995). The symbiosis between legume and rhizobium is responsible for a substantial part of global N flux in which atmospheric N_2_ is fixed to form ammonia, nitrate, and organic nitrogen. Rhizobial species (including the genera of *Mesorhizobium*, *Bradyrhizobium*, *Azorhizobium*, *Allorhizobium*, and *Sinorhizobium*) infect legume roots and induce the formation of root nodules (Stacey, 2007; Gopalakrishnan et al., 2015). The infection is initiated by the flavonoids released by legume hosts that induce the expressions of nodulation (*nod*) genes in rhizobia, which in turn trigger root cell divisions by producing lipo-chitooligosaccharide (LCO) signals (Dakora, 1995, 2003). Nodules can generally be classified into determinate and indeterminate ones. Determinate nodules are round shaped, with a well-defined homogeneous central fixation zone containing infected rhizobia-filled cells surrounded by uninfected cells (Schultze and Kondorosi, 1998). Indeterminate nodules have a gradient of developmental stages from the nodule tip to the root as a result of a persistent meristem that generates new cells continuously (Crespi and Gálvez, 2000), which is absent in determinate nodules. Well-studied model species with indeterminate nodules include *Medicago truncatula* (barrelclover), *Medicago sativa* (alfalfa), and *Pisum sativum* (pea), while *Glycine max* (soybean), *Vicia faba* (fava bean), and *Lotus japonicus* (birdsfoot trefoil) are typical model species for the study of determinate nodules (Gage, 2004).

Besides rhizobia, legumes form symbiosis with soil fungi such as arbuscular mycorrhizal fungi (AMF) and *Trichoderma* spp. (TR). The symbioses with AMF and TR are considered to be beneficial for plant growth (Brundrett, 1991; Woo et al., 2014). AMF colonize plant roots and radiate their hyphae into the surrounding soil, complementing the host’s root functions (Jansa et al., 2011). The enhanced root performance due to AMF has been shown in *G. max* (Wang et al., 2011), *V. faba* (Jia et al., 2004), *M. sativa* (Jansa et al., 2011), and *Phaseolus vulgaris* (common bean) (Tajini et al., 2012). Moreover, nodules normally have a high demand for inorganic phosphate (Pi) as nitrogenase functions in the bacteroid are highly ATP-consuming (Jakobsen, 1985; Liu et al., 2018). In such cases, AMF have the capability to accelerate the Pi uptake. The amount of Pi delivered to the plant varies with specific AMF (Ianson and Linderman, 1993; Valdenegro et al., 2001). On the other hand, TR-based biofertilizers have also been reported to enhance N, P, and K uptakes (Amaresan et al., 2020). TR communicates with the plant root by chemical signals such as auxins and small peptides. They could also colonize plant roots by penetrating the outer layers of the root tissue. One of the well-known beneficial effects of TR to plants is the solubilization of Pi by acidification, chelation or redox activities to improve the Pi availability to the plant. The benefits of AMF and TR to crop growth have been comprehensively reviewed (Szczałba et al., 2019).

Domestication is the conversion of wild plant species to cultivated ones through human selection and breeding of desirable characteristics over many generations, but it often results in the loss of genetic diversity (Gepts, 2010). It is well-documented that the domestication of legumes has emphasized the selection of favorable aboveground traits including larger seed size, palatability, reduced seed dormancy and heritability of other desirable agronomic traits (Abbo et al., 2014; Pérez-Jaramillo et al., 2016). Such a process of artificial selection has reduced the self-sustaining capability and increased sensitivities to diseases, abiotic stresses and nutrient depletion in cultivated legumes. Furthermore, artificial selection has also influenced underground traits including root architecture and root exudate composition, often unintentionally (Pérez-Jaramillo et al., 2016). This may affect the establishment of rhizospheric microbial communities in the soil where the legumes are grown. In this review, we will discuss how domestication and agricultural practices have influenced the interactions between legumes and rhizospheric microbes (rhizobia and fungi), with a focus on the potential impacts of such altered interactions on macronutrient acquisition by the host plant. We will also summarize the identified genes that regulate plant-rhizobia interactions in both plants and rhizobia, and the new study approaches using metabolic modeling and metabolic profiling, with the aim of contributing to a better understanding of legume-microbe symbiotic associations.

## Domestication and Agricultural Practices Influence the Diversity of Rhizobia and Soil Fungi

**FIGURE 1:**
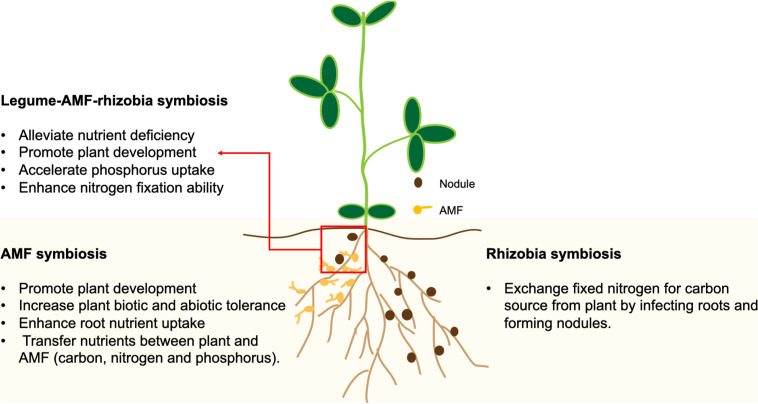
A schematic drawing representing the impacts of symbioses with arbuscular mycorrhizal fungi (AMF) and rhizobia on plant growth and nutrient uptake.

Legume-rhizobium mutualism contributes to the vast majority of non-anthropogenically fixed N in terrestrial ecosystems (Cleveland et al., 1999), and it is especially susceptible to anthropogenic environmental changes (Kiers et al., 2002). Through mutualistic symbiosis, legumes receive ammonia fixed from atmospheric N_2_ by rhizobia in the root nodule, in exchange for providing rhizobia with a carbon source obtained through photosynthesis (Figure 1). The efficiency and intensity of SNF is dependent on both rhizobia and plant hosts under a specific set of conditions (Lau et al., 2012). A study comparing the SNF efficiencies between wild (local) varieties and cultivars in major legume crops showed that the wild populations of alfalfa, pea and feugreek (*Trigonella foenumgraecum* L.) had better performance than their cultivated counterparts (Provorov and Tikhonovich, 2003). On the other hand, a study using wild and cultivated soybeans inoculated with the slow-growing *Bradyrhizobium japonicum* strain USDA110 or the fast-growing *Sinorhizobium fredii* strain CCBAU45436, indicated the SNF efficiency was improved by the domestication process (Muñoz et al., 2016). Moreover, the activity and diversity of rhizobia can be affected by human activities including the use of N fertilizer, cropping system and soil management system (Ferreira et al., 2000; Bizarro et al., 2011). The utilization of high levels of N fertilizer across soil ecosystems may result in the breakdown of nitrogen-fixing symbiosis between legumes and rhizobia (Foley et al., 2005). It was suggested that when the resources exchanged in mutualistic symbiosis are abundant in the soil and the host is less reliant on the microsymbiont, the plant-microbe interaction could shift from mutualism to parasitism (Bronstein, 2001). The genetic diversity of *rhizobium* in nodules of *P. vulgaris* was decreased partially due to the N fertilization (Caballero-Mellado and Martinez-Romero, 1999). A study showed that *Trifolium* species inoculated with long-term N fertilizer-treated rhizobial strains had less biomass and chlorophyll contents than plants inoculated with non-fertilizer treated rhizobia, implying an elevated N supply resulted in the evolution of less-mutualistic rhizobia that provide fewer growth benefits to plant hosts in the *Trifolium*-rhizobium symbiosis (Weese et al., 2015).

Arbuscular mycorrhizal fungi belong to the subphylum *Glomeromycotina*, which are asexual obligate biotrophs that infect roots and establish a mutualistic relationship with the host plant to complete their life cycle. The mutualism is characterized by the transfer of nutrients including P, N, and carbon (C) between the fungi and the plants (Figure 1). Moreover, AMF play an important role in improving N_2_ fixation by providing a favorable environment to facilitate the infection of plant roots by rhizobia (Mohammadi et al., 2012). It is believed that the responsiveness of crops to AMF is not under intentional selection by breeders during domestication (Bennett et al., 2013). However, agricultural activities such as soil disturbance, grazing, fertilizer application and monocropping may have reduced the AMF diversity and their community structure in the soil (Jansa et al., 2006). Plants having sufficient P supplies are also less responsive to AMF for symbiosis (Jansa et al., 2006). Moreover, soil types can also influence the structure of soil fungal communities (Chang et al., 2020). By simulating the monocropping of cultivated and wild soybeans in the greenhouse, it was shown that monocropping resulted in a stable fungal community in the rhizospheres of both types of soybeans, but with different community structures (Tian et al., 2020). Therefore, modern agricultural practices may have resulted in the reduced AMF diversity and the inadvertent selection of crop genotypes with reduced responsiveness to AMF for symbiosis (Bennett et al., 2013).

## Wild Legumes Recruit More Diverse Rhizobia and Soil Fungi

It is documented that domesticated legumes tend to have fewer compatible symbionts than wild legumes (Mutch and Young, 2004). Rhizobia varies on their ability to nodulate various legume species. Some strains show high nodulation specificities and only form nodules with a limited number of legume species, while others can have a wide range of hosts (Pueppke and Broughton, 1999). *G. max* is one of the most important cultivated legumes. *G. soja* is the wild progenitor species of *G. max*, with high genetic diversity and better resistance to environmental stresses (Muñoz et al., 2017). Both *G. soja* and *G. max* can be nodulated with rhizobia, including *Sinorhizobium fredii*, *Bradyrhizobium japonicum* and *Bradyrhizobium elkanii* (Wang et al., 2009). A study on bacterial isolates from *G. soja* nodules collected from different ecoregions in China showed that the biodiversity of rhizobia is associated with the geographical distribution of the particular ecotype of *G. soja* (Wu et al., 2011). Moreover, by comparing the rhizospheric bacteria between wild and cultivated soybeans in different soil types, wild soybean has the ability to recruit a higher abundance of *Bradyrhizobium* strains than cultivated soybean (Chang et al., 2019). A study of the impacts of domestication on the chickpea-*Mesorhizobium* symbiosis using 10 accessions of chickpea showed that the wild chickpea progenitor (*Cicer reticulatum*) could associate with more diverse *Mesorhizobium* populations than the cultivated chickpea (*C*. *arietinum*) (Kim et al., 2014).

By comparing the rhizospheric fungal communities of wild soybeans, ZYQ95 and 01-289, to those of cultivated soybeans, Williams 82 and Zhonghuang, the wild and cultivated germplasms were found to recruit different fungi in the rhizosphere (Chang et al., 2020). The wild soybeans recruited soil fungi with diverse potential functions while the cultivated soybeans mainly recruited those soil fungi which can enhance nutrient uptake by the plant (Chang et al., 2020). In another study conducted in the greenhouse, it was found that the genus *Paraglomus* was more enriched in the rhizosphere of wild soybean ZYQ95 while *Funneliformis* and *Rhisophagus* were more enriched in the rhizosphere of cultivated soybean Williams 82 (Zhang et al., 2019). In the field, no AMF could be found in the rhizosphere of Williams 82 while only *Paraglomus* was found in the rhizosphere of ZYQ95 under drought condition (Zhang et al., 2019). The reduced diversity of symbiotic rhizobia and fungi in modern legume cultivars compared to the wild relatives is likely a result of domestication. Agricultural practices such as monocropping might have played a role. Moreover, domesticated crops are usually grown in a confined cultivation area. As a result, the selection force on legume-associated soil microbe diversity in complex natural habitats might be absent.

## AMF Improve the Efficiency of Rhizobium-Mediated Nitrogen Assimilation in Both Wild and Domesticated Legumes

In a survey of 27 crops including legumes such as chickpea, soybean, grass pea, lentil, white clover and common bean, the domesticated crops and their wild relatives were inoculated with *Rhizophagus irregularis* (Blaszk, Wubet, Renker & Buscot) C. Walker & A. Schüßler strain EEZ 58 (Gi), which is a common strain of AMF in wild and agricultural lands. The inoculation led to increases in leaf Pi concentrations across all domesticated crops and wild relatives tested (Martín-Robles et al., 2018). In the same study, for the 14 non-leguminous crops such as barley, corn and tomato, when Pi availability in the soil was high, the growth benefits to the domesticated crops by the arbuscular mycorrhizae were reduced but not for their wild relatives (Martín-Robles et al., 2018). In another study, wild soybean (*G. soja*) or cultivated soybean (*G. max*) was inoculated with both *Scutellospora heterogama* (*S. heterogama*) and their own rhizobial cells (Eom et al., 1994). It was found that *S. heterogama* stimulated the triple symbiosis among the wild soybean plant, *S. heterogama* and the rhizobial cells (Eom et al., 1994; Figure 1). Furthermore, it was demonstrated that unimproved soybean accessions showed a greater mycorrhizal dependency (MD) than improved accessions (Khalil et al., 1994). When inoculated with the vascular-arbuscular mycorrhizal fungi, *Gigaspora margarita* or *Glomus intraradices*, under low soil Pi condition, the positive growth effects due to the inoculation were greater in the unimproved accessions than in the improved ones (Khalil et al., 1994). A follow-up study showed that the unimproved soybean accession having a high MD value, *G. soja* Sieb. & Zucc. PI 468916, developed symbiosis with *G. margarita* more quickly than *G. max* (L.) Merr. cv. Mandarin (unimproved) and *G. max* cv. Swift (improved), which have intermediate and low MD values, respectively (Khalil et al., 1999). Among the three soybean accessions, *G. soja* Sieb. & Zucc. PI 468916 also had higher phosphatase activities and higher percentage increases in phosphatase activities when inoculated with *G. margarita* (Khalil et al., 1999). In contrast, in a study on cowpea using three cultivated accessions, Katumani 80, KenKunde 1 and Kunde Mboga, and one wild accession, SP 219, it was found that the ability to respond to mycorrhizal inoculation was retained in the cultivated accessions, which are the common modern cultivars grown by smallholding farmers in Kenya (Oruru et al., 2017). In these experiments, the cowpea accessions were grown in sterilized soil inoculated with a filtrate from non-sterilized soil and a commercial mycorrhizal inoculum comprising *Funneliformis mosseae*, *Glomus aggregatum*, *Glomus etunicatum*, and *Rhizophagus irregularis* (Oruru et al., 2017). AMF root colonization was observed in all the accessions in both non-sterilized and sterilized soil. In all the accessions, the inoculation of the mycorrhizal inoculum improved the nodule number, dry weights of nodules, roots and shoots, and both the nitrogen and Pi levels in the shoots of all the wild and cultivated accessions. However, the increase in biomass of nodules, roots and shoots were more significant in the cultivated accessions than in the wild counterpart (Oruru et al., 2017). Katumani 80 and Kunde Mboga also had higher percentages of mycorrhizal colonization compared to the wild accession while Katumani 80 and KenKunde 1 had more nodules than the wild SP 219 (Oruru et al., 2017).

Although several studies showed that wild legumes benefited more from the inoculation with AMF than cultivated counterparts (Eom et al., 1994; Khalil et al., 1994, 1999; Martín-Robles et al., 2018), the opposite effect of AMF was also reported (Oruru et al., 2017). It is important to note that different legumes and AMF species were tested in these studies. It has been pointed out that the responses to soil fungi could be genotype-specific, instead of wild versus cultivated accessions of the legume in question (Prashar and Vandenberg, 2017). As discussed above, the response to soil fungi is probably not intentionally selected for during domestication. Therefore, it may not be appropriate to generalize the observations to all wild and domesticated legumes.

## The Capability to Respond to AMF and *Trichoderma* spp. (TR) Is Retained in Modern Legume Cultivars

During plant breeding, varieties which are more resistant to diseases are usually selected for. It has been hypothesized that such selections may also have influenced the susceptibility of these varieties to beneficial microbes such as AMF. When different varieties of chickpea were grown in paddock soil containing naturally occurring AMF, there was a correlation between the level of resistance to Phytophthora root rot (PRR) and the degree of AMF colonization on the root (Plett et al., 2016), where the PRR-susceptible variety, Sonali, had a significantly higher root AMF colonization level than the PRR-resistant varieties, Yorker and PBA HatTrick, although such a correlation was absent when the varieties were grown in sterilized soil inoculated with *Funneliformis mosseae* (Plett et al., 2016). A possible explanation for such a phenomenon is that there is a certain degree of overlap between genes which confer resistance against beneficial AMF and those against pathogenic microbes (Plett et al., 2016). Although there are few studies on the response of wild legumes to TR, current findings also suggest that the capability to respond to TR is retained in domesticated legumes. In a study on 23 wild and cultivated lentil accessions inoculated with commercial TR inocula, RootShield^®^ (RS) and RootShield^®^ Plus (RSP), based on *Trichoderma harzianum* T22 and *Trichoderma virens* G41, respectively, the wild accession, *Lens tomentosus* PI 572390, had enhancements in most of the tested agronomic traits such as root and shoot biomass (Prashar and Vandenberg, 2017). Positive effects on the cultivated accessions by such inoculations were also observed (Prashar and Vandenberg, 2017). However, in some accessions, be they cultivated or wild, the inoculation had no or negative effects on the same agronomic traits (Prashar and Vandenberg, 2017). This showed that the TR responses were genotype-specific rather than distinguished along the line of wild versus domesticated accessions (Prashar and Vandenberg, 2017).

It was hypothesized that new high-yield crops may be less responsive to AMF compared to their ancestors (Lehmann et al., 2012). However, a meta-analysis of the data on 320 different crops, including those in the families *Poaceae, Fabaceae, Pedaliaceae, Asteraceae*, *and Cucurbitaceae*, from 39 published studies suggested that the new crops have not lost the ability to respond to AMF compared to their ancestors (Lehmann et al., 2012). Wild and domesticated legume accessions usually have distinct root system architectures. For example, in a study on the quantitative trait locus (QTL) of root system architecture in soybean, a recombinant inbred (RI) population was produced by crossing a *G. max* parent, V71-370, with a *G. soja* parent, PI407162 (Prince et al., 2015). The domesticated parent *G. max* V71-370 had a larger root system than the wild parent. The resulting RI population displayed a transgressive segregation of the root traits, including taproot length and total root length, from the domesticated and wild parents (Prince et al., 2015). In another study on the QTL governing the root architecture of soybean, a cultivated parent, *G. max* Dunbar, and a wild parent, *G. soja* PI 326582A, were used to construct the RI population (Manavalan et al., 2015). The cultivated parent had longer tap root and more lateral roots than the wild parent (Manavalan et al., 2015). In common bean (*P. vulgaris*), it was found that the wild accessions, A1 and A2, have longer specific root lengths (root length: root dry weight) than the cultivated accessions, M1, M2, M3, M4, and M5, and a lower root density (root dry weight: root volume) than the cultivated M2 (Pérez-jaramillo et al., 2017). A link between domestication, specific root morphology and rhizobacterial community assembly was observed (Pérez-jaramillo et al., 2017). It is therefore convenient to suggest that the different root architecture of wild and domesticated legumes may result in different responses to soil fungi. However, based on the published data on root architecture and mycorrhizal growth responses, a meta-analysis suggested that there is no strong correlation between the mycorrhizal growth response and root diameter, root hair length or root hair density (Maherali, 2014). It appears that the differential responses to AMF colonization by different genotypes/accessions are influenced by factors other than the physical attributes of the root system. Similar to the improved efficiency of rhizobium-mediated nitrogen assimilation by AMF which happens in both wild and cultivated legumes, the capability to respond to AMF and TR is also retained in modern legume cultivars. As the symbiosis with AMF and TR are beneficial for plant growth (Brundrett, 1991; Woo et al., 2014), the capability of legumes to interact with these beneficial soil fungi should be have been selected by breeders during domestication.

## Functional Genes Involved in Legume-Rhizobium-AMF Symbiosis

Symbiosis with rhizobia and AMF play a key role in plant nutrient acquisition. The mechanisms of these interactions and the genes driving these processes have been investigated for decades. In these endeavors, mutant libraries have been highly valuable resources for the research on symbiotic nitrogen fixation in legumes. They have been used for the identification and functional analyses of essential SNF-related genes. Over the years, legume mutants have been produced using physical/chemical mutagenesis, insertional mutagenesis and targeted genome editing tools (Le Signor et al., 2009; Urnov et al., 2010; Cui et al., 2013), including zinc finger nucleases (ZFNs), transcription activator-like effector nucleases (TALENs) and CRISPR-Cas systems (Wang L. et al., 2017).

Genes and/or functions required for rhizobium and AMF symbiosis are summarized in Table 1. In *L. japonicus*, the biological functions of leghemoglobins were validated using RNAi and the CRISPR/Cas9 system (Ott et al., 2005; Wang et al., 2016, 2019). Leghemoglobins are plant hemoglobins required to maintain a low-oxygen environment inside root nodules for the efficient functioning of nitrogenases in bacteroids (Vázquez-Limón et al., 2012). The *Ljlb1*, *Ljlb2*, and *Ljlb3* mutants had elevated free-oxygen concentrations in infected zones and N_2_ fixation was abolished (Ott et al., 2005; Wang et al., 2019). Transcript analyses of bacteroid genes from the nodules of these mutants revealed lower expression levels of *nif* and *fix* genes compared to those from the wild type (Ott et al., 2005, 2009). In particular, the NifH protein was not detected despite the presence of *nifH* transcripts in the mutant nodules (Ott et al., 2005).

**TABLE 1:** Functional genes involved in legume symbiosis.

Gene	Function	Organism	References
*LjLB1*	Leghemoglobin	*Lotus japonicus*	Ott et al., 2005; Wang et al., 2016, 2019
*LjLB2*	Leghemoglobin	*Lotus japonicus*	Ott et al., 2005; Wang et al., 2016, 2019
*LjLB3*	Leghemoglobin	*Lotus japonicus*	Ott et al., 2005; Wang et al., 2016, 2019
*nifV*	Homocitrate synthase	*Azorhizobium caulinodans* ORS571 and *Bradyrhizobium*	Nouwen et al., 2017
*Fen1*	Homocitrate synthase	*Lotus japonicus*	Imaizumi-Anraku et al., 1997
*Rj4*	Symbiotic partnership specificity	*Glycine max*	Hayashi et al., 2014; Tang et al., 2016
*MtNFS1*	Symbiotic partnership specificity	*Medicago truncatula* Jemalong A17	Wang Q. et al., 2017; Yang S. et al., 2017
*MtNFS2*	Symbiotic partnership specificity	*Medicago truncatula* Jemalong A17	Wang Q. et al., 2017; Yang S. et al., 2017
*SYMRK*	Infection thread initiation and nodule development by rhizobia	*Lotus japonicus*	Kistner et al., 2005
*CASTOR*	Infection thread initiation and nodule development by rhizobia	*Lotus japonicus*	Kistner et al., 2005
*POLLUX*	Infection thread initiation and nodule development by rhizobia	*Lotus japonicus*	Kistner et al., 2005
*SYM3*	Infection thread initiation and nodule development by rhizobia	*Lotus japonicus*	Kistner et al., 2005
*SYM6*	Infection thread initiation	*Lotus japonicus*	Kistner et al., 2005
*SYM15*	Infection thread initiation and nodule development by rhizobia	*Lotus japonicus*	Kistner et al., 2005
*SYM24*	Infection thread initiation and nodule development by rhizobia	*Lotus japonicus*	Kistner et al., 2005
	Leghemoglobin	*Medicago truncatula*	Liese et al., 2017
	Nicotianamine synthase-like protein	*Medicago truncatula*	Liese et al., 2017
	Nodule-specific cysteine-rich peptides	*Medicago truncatula*	Liese et al., 2017
*GmEXLB1*	Regulation of root development and responses to abiotic stress	*Glycine max*	Kong et al., 2019
*GmACP1*	Acid phosphatase	*Glycine max*	Zhang et al., 2014

Efficient nitrogenase activities also depend on other conditions besides the microaerobic environment (Kuzma et al., 1993). Homocitrate is a component of the iron-molybdenum cofactor of nitrogenases (Rubio and Ludden, 2008). For rhizobia lacking the homocitrate synthase-encoding gene (*nifV*), homocitrate supplied by the host plant is a key element of successful SNF, as demonstrated using an SNF-defective *L. japonicus* mutant, *fen1* (Imaizumi-Anraku et al., 1997). A homocitrate synthase gene, *FEN1*, in the host plant was identified as responsible for compensating for the lack of *nifV* in rhizobia (Hakoyama et al., 2009). Additionally, an external supply of homocitrate was shown to restore the nitrogen-fixing capacity in the ineffective nodules.

On the other hand, the editing of genes responsible for the recognition mechanism underlying legume-rhizobium specificity enables nodulation with unspecific strains. In soybean, the disruption of the dominant allele *Rj4* allowed nodulation with some strains of *Bradyrhizobium japonicum* and *Bradyrhizobium elkanii* (Hayashi et al., 2014; Tang et al., 2016). *Rj4* encodes a thaumatin-like protein that usually restricts nodulation with these ineffective rhizobial strains. In *M. truncatula* Jemalong A17, the editing of two nodule cysteine-rich (NCR) peptide-encoding genes, *MtNFS1* and *MtNFS2*, allowed nodulation by *S. meliloti* strain Rm41 (Wang Q. et al., 2017; Yang S. et al., 2017). NCR peptides control and regulate the symbiotic partnership specificity in *M. truncatula*. *MtNFS1* and *MtNFS2* were identified using a recombinant inbred line (RIL) population of Jemalong A17 and DZA315. Parental genotypes were segregated according to their ability to form functional nodules with *S. meliloti* strain Rm41 or not (Wang Q. et al., 2017; Yang S. et al., 2017).

Seven *L. japonicus* genes (*SYMRK*, *CASTOR*, *POLLUX*, *SYM3*, *SYM6*, *SYM15*, and *SYM24*) are required for infection thread initiation for both fungal and bacterial symbiosis (Kistner et al., 2005). Mutant phenotypes showed that these genes are involved in the intracellular infection by fungal and bacterial symbionts. Additionally, these genes are required for nodule development by rhizobia, specifically for the formation of nodule primordia, except *SYM6*, since small nodule-like structures could still be observed on *sym6* mutants during the interaction with *Mesorhizobium loti* but not on the other six mutants. After inoculation with AMF, *sym* mutant roots had a limited ability to form arbuscules, indicating the key role of the symbiosis-related genes for intracellular infection by fungal partners (Kistner et al., 2005).

Symbiotic nitrogen fixation in nodules is also affected when the growth is slow due to nutrient deficiency. An RNA-Seq transcriptome profiling of the nodules of *M. truncatula* inoculated with *S. meliloti* under P-deficient conditions revealed lower nitrogenase activities (Liese et al., 2017). The reduction in SNF efficiency was due to a downregulation of genes encoding leghemoglobins, nicotianamine synthase-like proteins and NCR peptides. Also, low-Pi conditions reduced growth, nodule numbers and P concentrations in stem, root and nodule tissues compared to the control (Liese et al., 2017). However, the P concentration in nodules was relatively higher than in stem and root tissues. In soybean, *GmEXLB1*, encoding an expansin, was identified as a key gene involved in the response to low-P stress through transcription profiling (Kong et al., 2019). Expansins are involved in the regulation of root development and responses to abiotic stresses in plants (Cosgrove, 2015). It was observed that *GmEXLB1* expression was induced in the lateral roots of soybean under P starvation conditions. The ectopic expression of *GmEXLB1* in transgenic *Arabidopsis* promoted changes to the root architecture by increasing the number and length of lateral roots, thus improving P acquisition under low-P conditions (Kong et al., 2019). The acid phosphatase-encoding gene, *GmACP1*, has also been associated with P efficiency in soybean (Zhang et al., 2014). *GmACP1* is located in the QTL, *qPE8*, related to soybean P efficiency, on chromosome 8. Transgenic soybean hairy roots overexpressing *GmACP1* showed a 2.3-fold increase in acid phosphatase activity and increased the efficiency of P usage by 11.2-20.0% relative to the control (Zhang et al., 2014).

## Metabolic Modeling and Metabolic Profiling Studies on Legume-Rhizobium Symbiosis

Legume-rhizobium symbiosis involves biological nitrogen fixation as well as the exchange of nutrients. Transport systems and carbon-nitrogen metabolism are coordinated to provide nutrients for effective SNF. Each host plant-rhizobium interaction is association-specific and determines the accumulation of carbon and energy storage metabolites inside the microsymbionts (Terpolilli et al., 2012). Metabolic modeling provides new insights into the metabolism in plant systems. An emerging approach to studying the process of SNF in legumes is constraint-based modeling, which relies on network topology and the integration of genomic and high-throughput data for analyzing the metabolic capabilities of organisms (Feist et al., 2009; Heirendt et al., 2019). Among the most frequently used frameworks is flux balance analysis using genome-scale models (Orth et al., 2010). This approach has been successfully used to study the process of SNF carried out in mature nodules by rhizobia. Metabolic network reconstructions have been reported for *S. meliloti* (Zhao et al., 2012; diCenzo et al., 2016, 2018), *Bradyrhizobium diazoefficiens* (Yang Y. et al., 2017), the symbiotic forms of *Rhizobium etli* (Resendis-Antonio et al., 2007, 2012), and *Sinorhizobium fredii* (Contador et al., 2020). These studies included the nutrient requirements for each N_2_-fixing relationship and performed preliminary analyses of SNF such as flux analyses of relevant biochemical pathways (e.g., the TCA cycle, the pentose phosphate pathway, and the production of cofactors for nitrogenase) and determination of the genes required for an efficient SNF through computational simulations. However, these analyses simplified the plant metabolism by only including inputs from the host plant to the microsymbiont metabolism during symbiosis.

Recently, the impact of wild and cultivated soybeans on nitrogen fixation was assessed in the *S. fredii* model (Contador et al., 2020). Transcriptome profiles of *S. fredii* in symbiotic conditions were used to capture the differential nitrogen-fixing capacity of *S. fredii* strain CCBAU45436 in symbiosis with *G. max* C08 and *G. soja* W05, respectively. The bacteroid model quantified and predicted a higher nitrogen fixation activity and C/N ratio of *S. fredii* with the cultivated soybean than with the wild accession. This is consistent with previous experimental observations that this soybean cultivar (C08) outperformed the wild accession (W05) in a series of nitrogen fixation-related traits including nodule number, total nitrogen and total ureide accumulation, and new QTLs for ureide content and nodule fresh weight have been identified using RIL populations (Muñoz et al., 2016). An examination of these QTLs revealed a very low diversity in some regions among cultivated soybeans. Domestication process was suggested as the responsible for the selection of these traits (Muñoz et al., 2016).

The representation of whole-rhizobium metabolic networks has been achieved for *S. meliloti* and *B. diazoefficiens* USDA110 (diCenzo et al., 2016; Yang Y. et al., 2017). These reconstructions were used to build context-specific models to characterize the metabolic capabilities of rhizobia in bulk soil, rhizosphere and nodule bacteroids. However, metabolic models have been published for only two legume species, *G. max* and *M. truncatula*, despite the significance of legumes to sustainable agriculture. A compartmentalized reconstruction for *G. max* was used to construct a multi-organ model to represent the reserve mobilization in the cotyledon and hypocotyl/root tissues (Moreira et al., 2019). This reconstruction can also be used to represent other tissues and conditions since the whole set of metabolic reactions in soybean were used to construct the metabolic model. Annotations of the *G. max* genome were used to define the gene-protein-reaction associations in the model. On the other hand, the *M. truncatula* model was used to capture both biomass production during day and night conditions and host-microsymbiont interactions (Pfau et al., 2018). This was the first attempt to elucidate the effects of symbiosis on metabolic fluxes and plant growth. Computational analyses revealed the costs and benefits of the symbiotic system for biomass growth when external ammonium is not available. Recently, the *M. truncatula* model was built to represent multi-organism metabolic interactions in different developmental zones within the nodule during nitrogen fixation (diCenzo et al., 2020). The multi-organism model consisted of *M. truncatula* nodulated by *S. meliloti* Rm1021. The plant system was represented by a multi-compartmental reconstruction that includes root and shoot tissues together with five nodule zones (apical meristem, distal, proximal, interzone, and nitrogen fixation zones). The accuracy of the metabolic reconstruction for *S. meliloti* was improved over the previous versions of the model. The integrated model was used to evaluate the use of nutrients by N_2_-fixing bacteroids, N_2_ fixation efficiency and costs related to the nitrogen fixation process.

The metabolic states of the legume-rhizobium association can also be assessed by performing metabolic profiling (Roessner et al., 2001; Rambla et al., 2015), which can provide a comprehensive picture of a particular sample or a genetically manipulated system. Metabolic profiling has been used to study metabolic shifts in SNF associations. Recently, metabolic profiling were performed on effective and ineffective nodules of soybean to identify the active processes according to the partners involved in the symbiosis (Agtuca et al., 2020). Metabolite levels were determined to characterize the different plant phenotypes and interactions with rhizobia. Similar comparative studies have been performed in *Medicago* and *L. japonicus* to investigate metabolites and related pathways that influence nodule metabolism during nitrogen fixation (Desbrosses et al., 2005; Ye et al., 2013; Gemperline et al., 2015). Other metabolomic studies have characterized the differentiation process from free-living rhizobia into bacteroids to detect differences in the metabolite composition of these two physiological conditions (Vauclare et al., 2013). Metabolite profiling of root hairs was also performed to characterize the early stage of rhizobial infection, by identifying those metabolites the accumulation of which was regulated in response to rhizobial inoculation (Brechenmacher et al., 2010).

## Conclusion and Perspective

Legumes engage in mutualistic relationships with rhizobia and mycorrhizal fungi, enabling them to obtain essential nutrients, promote growth, and enhance biotic and abiotic stress resistance. There is extensive evidence to support that AMF affect the establishment and functions of rhizobial nodulation in both wild and cultivated legumes and enhance nitrogen acquisition in the host plant through SNF. However, domestication has reduced the genetic diversity in host plants and impacted their compatibility with rhizobial strains and AMF. And the intensive applications of chemical fertilizers and large-scale monoculture farming practices during domestication actually have a negative impact on AMF community structures and their colonization of legume roots. It is important for researchers to engineer or screen for rhizobium strains, which are both competent for nodulation and capable of high nitrogen fixation efficiency, and AMF, which can effectively improve the nutrient acquisition by the host plants. Meanwhile, it is also vital to improve host plant’s ability to interact with the best beneficial mutualistic symbionts. Understanding the genetic control of the symbiosis specificity between wild and cultivated legumes and identifying the key genetic factors controlling the symbiotic interaction will provide useful information for improving legume-soil microbe symbiosis. Moreover, utilizing advanced approaches such as metabolic modeling and metabolic profiling to investigate the molecular mechanisms of metabolite exchanges among legumes, AMF and rhizobia under nutrient-deficient conditions will help researchers better understand legume-microbe symbiosis, and ultimately contribute to the implementation of sustainable agriculture.

## Author Contributions

H-ML and AL planned and coordinated the writing and finished the final draft. AL and Y-SK put together the first complete draft. CC contributed to the literature search and writing. All authors contributed to the article and approved the submitted version.

## Conflict of Interest

The authors declare that the research was conducted in the absence of any commercial or financial relationships that could be construed as a potential conflict of interest.
